# Transcriptional and Physiological Analyses to Assess the Effects of a Novel Biostimulant in Tomato

**DOI:** 10.3389/fpls.2021.781993

**Published:** 2022-01-11

**Authors:** Maria Cristina Della Lucia, Ali Baghdadi, Francesca Mangione, Matteo Borella, Walter Zegada-Lizarazu, Samathmika Ravi, Saptarathi Deb, Chiara Broccanello, Giuseppe Concheri, Andrea Monti, Piergiorgio Stevanato, Serenella Nardi

**Affiliations:** ^1^Department of Agronomy, Food, Natural Resources, Animals and Environment, University of Padua, Padua, Italy; ^2^Department of Agricultural and Food Sciences, University of Bologna, Bologna, Italy; ^3^Sipcam Italia S.p.A. Belonging Together With Sofbey SA to the Sipcam Oxon S.p.A. Group, Pero, Italy

**Keywords:** plant biostimulant, functional characterization, drought stress, tomato, transcriptomics, differentially expressed genes, physiological traits

## Abstract

This work aimed to study the effects in tomato (*Solanum lycopersicum* L.) of foliar applications of a novel calcium-based biostimulant (SOB01) using an omics approach involving transcriptomics and physiological profiling. A calcium-chloride fertilizer (SOB02) was used as a product reference standard. Plants were grown under well-watered (WW) and water stress (WS) conditions in a growth chamber. We firstly compared the transcriptome profile of treated and untreated tomato plants using the software RStudio. Totally, 968 and 1,657 differentially expressed genes (DEGs) (adj-*p*-value < 0.1 and |log2(fold change)| ≥ 1) were identified after SOB01 and SOB02 leaf treatments, respectively. Expression patterns of 9 DEGs involved in nutrient metabolism and osmotic stress tolerance were validated by real-time quantitative reverse transcription PCR (RT-qPCR) analysis. Principal component analysis (PCA) on RT-qPCR results highlighted that the gene expression profiles after SOB01 treatment in different water regimes were clustering together, suggesting that the expression pattern of the analyzed genes in well water and water stress plants was similar in the presence of SOB01 treatment. Physiological analyses demonstrated that the biostimulant application increased the photosynthetic rate and the chlorophyll content under water deficiency compared to the standard fertilizer and led to a higher yield in terms of fruit dry matter and a reduction in the number of cracked fruits. In conclusion, transcriptome and physiological profiling provided comprehensive information on the biostimulant effects highlighting that SOB01 applications improved the ability of the tomato plants to mitigate the negative effects of water stress.

## Introduction

Biostimulants are increasingly important in agriculture, being considered environmentally sustainable and economically favorable answers to optimize crop productivity (Rouphael and Colla, 2020). There are currently several definitions of biostimulants. Conceptually they can be defined as non-nutrient substances or microorganisms applied to plants to promote plant growth, nutrient use efficiency, and stress tolerance (Calvo et al., 2014; Du Jardin, 2015).

Their action on plants is exerted through several mechanisms among which are the capacity to produce a hormone-like activity, the enhancement of photosynthesis, and the promotion of the activity of plant-soil microorganisms (Nardi et al., 2016; Van Oosten et al., 2017; Hellequin et al., 2020; Della Lucia et al., 2021).

Biostimulants are derived from a broad variety of compound classes that include mainly humic and fulvic substances, seaweed extracts, beneficial microorganisms, protein hydrolyzates and other nitrogen-containing compounds, carbohydrates, and inorganic compounds (Rouphael and Colla, 2020).

They are increasingly studied and used to mitigate the negative effects of environmental stresses such as lack of water and nutrients on cultivated plants (Van Oosten et al., 2017). Drought stress is one of the major problems of crops and especially limited water availability is a frequent suboptimal condition encountered by horticultural crops as tomato (Bulgari et al., 2019). One of the main biochemical impairing conditions occurring in plants under drought stress is the oxidative damage brought on by the overproduction of reactive oxygen species (ROS) (Fahad et al., 2017). The physiological responses induced by water stress include decreased cell turgor (Le Gall et al., 2015), leaf rolling (Kadioglu et al., 2012), inhibited CO_2_ exchange, decreased photosynthetic efficiency and chlorophyll contents (Mao et al., 2015), and, finally, a drop in overall crop performance.

The diverse nature of many biostimulants and the wide variety of their constituents are adding complexity to the processes of modes of action discovery, product description, production, legislation, and use (Yakhin et al., 2017). Owing to the advancements in omics sciences, relevant steps forward have been made in the last years in studying the modes of action of plant biostimulants (Ertani et al., 2009; Przybysz et al., 2014; Colla et al., 2017a; Lucini et al., 2020). The joint use of omics technologies, such as transcriptomics, metabolomics, and phenomics, can comprehensively clarify the biological basis underlying the biostimulation activity. Moreover, product screening strategies using omics technologies are considered efficient and cost-effective for developing and testing biostimulant substances (Paul et al., 2019).

The mRNA sequencing technology has become a crucial tool for differential gene expression analysis. It is advantageously used to monitor plant status and it has been adopted to study the biostimulants’ function in several works in a wide variety of crop species and biostimulant compounds in different environmental conditions (Briglia et al., 2019; González-Morales et al., 2021). Plant gene expression is dependent upon a multitude of factors. It is regulated during plant development, and it changes in response to environmental factors, and abiotic or biotic stresses (Costa-Silva et al., 2017).

To thoroughly study the biostimulant effects on plants, the combination of transcriptome profiling with plant phenomics, which measures specific physiological parameters, has been exploited and suggested (Briglia et al., 2019). The use of chlorophyll fluorescence images combined with phenotyping structures enables rapid screening of the overall photosynthetic performance and characterization of a plant’s potential to harvest light energy, which is related to biomass formation and plant structure (Tschiersch et al., 2017). Photosynthesis prediction is the first step to preannounce crop growth, yield, and quality in response to environmental changes (Zhang et al., 2018) and predict the onset of abiotic stresses (Murchie and Lawson, 2013).

This work aimed to study the effects in tomato of foliar applications of a novel calcium-based biostimulant (SOB01) in well-watered and water scarcity conditions by means of an omics approach involving transcriptomics and physiological profiling. A calcium-chloride fertilizer (SOB02) was used as a product reference standard.

Firstly, we analyzed the transcriptome profiling mRNA sequencing of treated and untreated tomato plants after the first treatment application, at one developmental stage (5th inflorescence, BBCH65). We then selected nine mRNA transcripts and we evaluated their expression patterns by real-time qPCR on plants grown under well-watered and water stress conditions at three different plant phenological stages (BBCH65, BBCH75, and BBCH85) to evaluate the effects of the treatments in two different water regimes. At the same time, we conducted physiological evaluations to functionally validate the selected transcripts potentially relevant for plant growth and yield and to describe the plant physiological responses.

## Materials and Methods

### Plant Material, Growing Conditions, and Experimental Setup

Tomato (*Solanum lycopersicum* L.) seeds, var. Micro-Tom, were provided by Sipcam Oxon S.p.A. Seedlings were grown individually in 1.2 L pots filled with a mixture of 90% peat (white sod peat (10–25 mm), white peat (0–25 mm), and peat fiber) and 10% perlite with a concentration of N (140 mg L^–1^), P (160 mg L^–1^ P_2_O_5_), K (1,680 mg L^–1^ K_2_O), Mg (100 mg L^–1^), and all necessary trace elements. Pots were placed within a growth chamber under controlled environmental conditions of 20–24°C temperature, 60% relative humidity, and LED lighting for 12 h/day. A soluble commercial fertilizer with 20% N, 20% P, 20% K content by weight was added twice a week to each pot. A randomized complete block design with two blocks was set up with two water regimes and two foliar treatments in a factorial combination. A total of 30 plants including 6 biological replicates (plants) for each experimental condition were used. The two applied treatments were a novel calcium-based mixture with a concentration of 5 ml L^–1^ (SOB01) and a calcium-chloride solution with a concentration of 10.05 gr L^–1^ (SOB02). These products were provided by Sipcam Oxon S.p.A. and the composition of SOB01 is described in Supplementary Table 1. Each solution was diluted in ultra-pure water and was applied as a foliar spray, at a volume of 10 ml per plant at three different phenological stages BBCH65 (5th inflorescence), BBCH75 (5th fruit cluster), BBCH85 (50% of fruits show typical fully ripe color). Rates of application of both SOB01 and SOB02 were defined to achieve the same amount of Ca per hectare following the label recommendations in three key developmental stages of tomato crop. The two water regimes applied were well-watered at pot water capacity (WW) and water stress, 65% of pot water capacity (WS). Water stress was induced before the flowering stage, after 2 weeks from transplant for those plants subjected to drought stress and kept throughout the experiment. The water content of the pots was continuously measured through an automatic moisture content monitoring system of independent loading cells and data were recorded as hourly average in a data logger (Gmr Strumenti Sas, Italy).

### Transcriptome Profiling

#### Sample Harvest

For transcriptome sequencing well-watered plants were sampled just before (untreated, *t* = 0) and after 48 h from treatment application (treated) at BBCH65. The choice of this specific sampling time was based on previous similar experiments that allowed the detection of molecular responses in the early hours following a foliar treatment. Each plant sample was made by two leaf disks collected per single plant. Three biological replications were analyzed for each entry. For gene expression analysis through quantitative RT-PCR, we collected four sample replicates (2 leaf disks per single plant) just before and 48 h after each treatment application at the three developmental stages previously described (BBCH65, BBCH75, and BBCH85), from well-watered and water-stressed plants. Samples were immediately stored at −80°C until RNA extraction.

#### Direct mRNA Isolation

mRNA sequencing was carried out at the phenological stage BBCH65 (5th inflorescence) before and after 48 h of treatment application. mRNA was directly isolated using the Dynabeads mRNA Direct Micro Kit (Thermo Fisher Scientific, Carlsbad, CA, United States) following the protocol for mRNA isolation from tissues. Briefly, we ground 30 mg of frozen leaf samples for 3 min with the Tissue Lyser (Qiagen, Germany) together with 100 μl of lysis-binding buffer. The lysates were then combined with 20 μl of pre-washed Dynabeads Oligo (dT) and mixed by pipetting up and down three times. The sample tubes were placed in a mixer for 5 min to allow the mRNA to anneal to the Dynabeads Oligo (dT) and successively placed in DynaMag-2 Magnet (Thermo Fisher Scientific) for 1 min to discard the supernatant. Samples were then removed from the magnet and the Dynabeads-mRNA complex was resuspended in 100 μl of Washing Buffer A. Again, the supernatant was removed by placing the sample tubes in the DynaMag-2 Magnet and this step was repeated. 100 μl of Washing Buffer B was added to the remaining Dynabeads-mRNA complex and washed two times by discarding the supernatant using the DynaMag-2 Magnet. Finally, the Dynabeads-mRNA complex was eluted in 20 μl of ice-cold 10 mM Tris–HCl and incubated at 65–80°C for 2 min. Once the tubes were placed in the magnetic rack, we transferred the supernatant containing the purified mRNA into a new tube and its quality and quantity were checked by Agilent TapeStation 1500 (Agilent Technologies Inc., Santa Clara, CA, United States). The average mRNA yields obtained were 2,150 ± 479 pg/μl. Once extracted, the quantification method showed contamination from 18S or 28S sequences that were removed to avoid unwanted amplicons by performing an additional washing step at the end of the mRNA extraction protocol.

#### Sequencing Library Preparation

Sequencing libraries were prepared using Ion Total RNA-Seq Kit v2 (Thermo Fisher Scientific). mRNA was fragmented with Rnase III and the reaction was assembled as follows: 10 μl of poly(A) RNA, 1 μl of 10× Rnase III Reaction Buffer, and 1 μl of Rnase III. The incubation was done in a thermal cycler at 37°C for 3 min. Immediately after incubation, we added 20 μl of nuclease-free water to stop the reaction. After fragmentation, we proceeded to purify the 32 μl fragmented RNA by adding 5 μl of beads, 90 μl of Binding Solution Concentrate and 150 μl of 100% ethanol. After 5 min of incubation, samples were placed in the magnetic rack for 6 min to separate the beads from the solution, then we discarded the supernatant. Beads were washed with 150 μl of Wash Solution Concentrate for 30 s and the supernatant was discarded. We eluted the RNA from the beads by adding 12 μl of pre-warmed (37°C) nuclease-free water to each sample. At the end of the purification steps, we quantified the fragmented RNA with Agilent TapeStation 1500 (Agilent Technologies). The second step involved the hybridization and ligation of RNA. The hybridization master mix was prepared as follows: 3 μl of fragmented RNA, 2 μl of Ion Adapter mix v2, and 3 μl of Hybridization solution. The thermal cycler was set at 65°C for 10 min and 30°C for 5 min. The ligation master mix was composted by 8 μl of hybridization reactions, 10 μl of 2× ligation buffer, and 2 μl of ligation enzyme mix and incubated 30°C for 1 h. Then, we performed the reverse transcription with 2 μl of nuclease-free water, 4 μl of 10× RT buffer, 2 μl of 2.5 mM dNTP Mix, 8 μl of Ion RT Primer v2, together with 20 μl of the ligation reaction. The reaction was incubated at 70°C for 10 min, then added 4 μl of 10× SuperScript III Enzyme Mix and incubated again at 42°C for 30 min. cDNA was purified with the same procedure described above. The third step involved the amplification of cDNA with the following mix: 6 μl of cDNA, 45 μl of Platinum PCR SuperMix High Fidelity, and 1 μl of Ion Xpress RNA 3′ Barcode Primer. The reaction was set at 94°C for 2 min, 2 cycles at 94°C for 30 s, 50°C for 30 s, 68°C for 30 s, then 16 cycles at 94°C for 30 s, 62°C for 30 s, 68°C for 30 s, and the final hold at 68°C for 5 min. The amplified cDNA was purified as described above, eluted with 15 μl nuclease-free water, quantified through D1000 screen Tape (Agilent TapeStation 1500), normalized, and pooled.

#### mRNA Sequencing

The sequencing run was performed using an Ion Torrent S5 (Thermo Fisher Scientific) with Ion 540 kit OT2. 8 μl of the 100 pM cDNA library were diluted in 100 μl of nuclease-free water and used to prepare the template positive Ion sphere particles with the Ion One Touch 2 instrument (Thermo Fisher Scientific). The template positive Ion sphere particles were used for the enrichment process with the Ion ES instruments (Thermo Fisher Scientific). Samples were then sequenced with Ion 540 chip kit (Thermo Fisher Scientific).

#### Sequencing Data Analysis

Raw RNA-Seq reads were filtered to remove low-quality reads (phred-like *Q*-value ≤ 20). Filtered reads were mapped to *S. lycopersicum* genome (SLv3.0) (publicly available from NCBI, GenBank accession GCA_000188115.3) using Bowtie2 (v2.4.2) (Langmead and Salzberg, 2012). Mapped files were processed using samtools (v1.11) (Li et al., 2009) and raw read counts were counted for all predicted genes using bedtools multiBamCov (Quinlan and Hall, 2010). To remove less informative data, we filtered genes with an overall expression level smaller than 20. DESeq2 R package (v.1.30.0) (Love et al., 2014) was used to perform the inferential analysis and obtain differentially expressed genes (DEGs) across the biological conditions. An adjusted *p*-value < 0.1 and a range of log2-fold change ≥ 1.0 to ≤ –1.0 were set as thresholds of significance to select DEGs. Genes with a log2-fold change > 1 were regarded as up-regulated DEG, while genes with a log2-fold change < −1 were regarded as down-regulated DEG. DEGs were then subjected to enrichment analysis of Gene Ontology (GO) terms at an FDR threshold of 0.05 using ShinyGO v0.66^1^ (Ge et al., 2020) to functionally categorize the genes by Biological Process, Cellular Component, and Molecular Functions.

### Real-Time Quantitative Reverse Transcription PCR

Nine gene sequences were selected for further characterization of their expression on the three timings of treatment application and in WW and WS conditions. The genes annotations and primers sequences used in this study are reported in Table 1. Primer design with Primer Express V3.0 (Thermo Fisher Scientific), was done starting from mRNA sequences downloaded from Tomato Genome SLv3.0. The gene sequences belonged to the following significantly enriched functional categories: protein serine/threonine kinase activity, ion transmembrane transporter activity, response to stress, response to stimulus, and RNA binding. All these categories have emerged as relevant in the in-depth study of biostimulant applications (Ertani et al., 2017; Barone et al., 2019). Real-time quantitative reverse transcription PCR (RT-qPCR) amplification and detection were conducted on a Quant Studio 12K Flex Real-Time PCR (Thermo Fisher Scientific) using qPCRBIO SyGreen 1-step kit (Resnova-PCR Biosystem, Rome, Italy). The 10 μl of reaction mixture contained 5 μl of SYBR Green, 0.5 μl retrotranscriptase, 0.4 μl of forward and reverse primers, 0.7 μl of nuclease-free water, and 1 μl of RNA. The threshold cycle (Ct) values obtained were normalized against the average transcript abundance of three housekeeping genes (GAPDH *Solyc05g014470*, Actin *Solyc11g005330*, and UBI *Solyc01g056940*), using the formula: 2^–(ΔCt)^ in which ΔCt is obtained from the difference between the Ct of the target gene and the Ct of the control gene (Livak and Schmittgen, 2001; Schmittgen and Livak, 2008).

**TABLE 1:** Selected genes used for validation of RNA-Seq data using real-time quantitative RT-PCR.

Gene ID	Gene name	Gene description	Gene ontology term	Forward Primer 5′,3′ and Reverse Primer 3′,5′
*Solyc02g090510*	*CRK*	CDPK-related protein kinase	GO:0004683–calmodulin-dependent protein kinase activity	AATGCCAGCACTAATTCTACTC CCCTCTTCCAACTTCCTCTC
*Solyc01g080300*	*ABCI12*	ATP binding cassette	GO:0009236–cobalamin biosynthetic process	TCTTTTCTCCTCTTCTTCCTCC ACGACTTCAATGCTCATCAC
*Solyc06g071500*	*SlBOR02*	Boron transporter	GO:0080139–borate efflux transmembrane transporter activity	AGAGGAGAAAGAAGCCCCAG AGACACACAAACAAGGAAACAC
*Solyc01g103890*	*MRS2-4*	Magnesium transporter	GO:0015095–magnesium ion transmembrane transporter activity	TCCCTTTTCGTTTTTTCCCC TTCCCCATCTTACCCAGTTC
*Solyc01g067740*	*SODCC1*	Superoxide dismutase [Cu-Zn]	GO:0004784–superoxide dismutase activity	CTATTACCGACAAGCAGATTCC AATACCACAAGCAATCCTTCC
*Solyc03g006680*	*5203_PHOS32*	Universal stress protein	GO:0006950–response to stress	ACTCAATAAGTCCCAACTCCC TTCTACCACCAACCATCCC
*Solyc07g053200*	*3369_PHOS32*	Universal stress protein	GO:0006950–response to stress	CGTCCAAAACTACCTCCGTC TCAATCTCAACCTCTCCACTTC
*Solyc10g079820*	*ERD15*	Dehydration-induced protein	GO:0005515–protein binding	ACCCAAATACTTTGAGAAGCC TGACACCTACCTTGCTCTATAC
*Solyc12g056790*	NAC1	NAC1 stress-related	GO:0045449–regulation of transcription	AACCTCTCTCTACATCCATACC GAAACTAACCTCCAACCAACC

### Physiological Analysis

Every physiological measurement was carried out during the experiment before, and 48 h after the applications of the two products at the three aforementioned phenological phases to detect early physiological responses induced by the treatments’ application.

#### Dry Matter and Fruit Cracking Measurements

Tomato fruits were harvested when fruits reached the maturity stage. At harvest fruit’s fresh and dry weights were recorded for each plant. The dry matter (DM) of tomato fruit was measured by oven-drying a sub-sample at 60°C until a constant weight was obtained. At the harvest time, the number of cracked tomato fruits was counted, and cracking rates were calculated.

#### Gas Exchange Analysis

Gas-exchange measurements were taken with an infrared gas analyzer (CIRAS-3, PP Systems, Amesbury, MA, United States). The rates of net photosynthesis (Pn) and stomatal conductance (gs) were measured on the youngest fully expanded leaf in all the plants. The leaf was marked and the measurement after the treatment application occurred on the same leaf within the same treatment event. A different leaf was selected in every biostimulant application time due to plant growth. Measurements were made under saturating light at a PPFD (photosynthetic photon flux density) of 1,500 μmol m^–2^ s^–1^ with 390 μmol mol^–1^ of CO_2_ flux density surrounding the leaf. The leaf cuvette had a 2.5 cm^2^ window, and the light was provided by red, green, and blue light-emitting diodes. Leaf temperature for all measurements was kept at ambient temperature.

#### Chlorophyll Fluorescence Measurements

Chlorophyll fluorescence measurements were estimated with a Hansatech Handy Plant Efficiency Analyzer (Handy PEA, Hansatech Instruments, King’s Lynn, Norfolk, United Kingdom) on two intact leaves per pot before every treatment application and after approximately 48 h. The Handy-PEA sensor was placed on the leaf clip with the shutter open. The leaves on which the measurements were carried out were similar to the ones used for photosynthesis measurements. The leaf changed among application times due to plant growth. The saturated light level of the instrument was set at 3,000 μmol m^–2^ s^–1^ to generate a true fluorescence intensity of maximum value. Key fluorescence parameters were analyzed: the minimum fluorescence (F_0_, dark-adapted leaf pre-photosynthetic fluorescent state), the maximum fluorescence (Fm, measured under a pulse of super-saturating light after the leaves were dark-adapted), and the fluorescence variable (Fv) which is the variable component of fluorescence obtained by the difference between Fm and F_0_. The ratio Fv/Fm is proportional to the quantum yield of photochemistry in photosystem II (PSII) and shows a high degree of correlation with the quantum yield of net photosynthesis.

#### Soil Plant Analytical Division Measurements

The leaf chlorophyll content index was determined using a Soil Plant Analytical Division (SPAD) chlorophyll meter (SPAD 502, Konica Minolta Sensing, Inc., Ramsey, NJ, United States). It calculates the SPAD value based on the intensity of light transmitted around 650 nm (red band), where absorption by chlorophyll is high, and a reference wavelength around 940 nm (Markwell et al., 1995). Measurements took place on two fully expanded leaves per plant selected one at half-height of the plant and one among the uppermost leaves.

### Statistical Analyses

Analysis of variance (ANOVA) of the data collected on physiological parameters of plants was performed using Statistix 10 (Analytical Software, Tallahassee, FL, United States).

Data analysis of genes expression levels was conducted using Statistica v13.4 (Dell, Round Rock, TX, United States). Statistically significant differences between the mean values of SOB01 and SOB02 treated plant samples were determined using the *t*-Student test (*p* < 0.05).

## Results

### Transcriptomics Analysis

#### Direct mRNA Isolation and Library Preparation

The mean mRNA content of the extracted samples was 2,150 ± 479 pg/μl. The fragmentation step produced sequences of an average length of about 160 bp as represented in Supplementary Figure 1C. The library preparation protocol allows obtaining a quantity of amplified cDNA of approximately 15,000 pg/μl. Supplementary Figure 1D reports the final quantification of the cDNA library obtained from a tomato leaf sample with a read length ranging from 100 to 700 bp and a mean of 200 bp.

#### Sequencing and Data Analysis

Sequencing data were downloaded from the Torrent server which provides a preliminary run and samples quality check. The information available is the chip loading density, the percentage of loading, enrichment, clonal sequence, and final library (Supplementary Figure 2). In total, 139,979,951 single-end sequences were obtained across all samples. Sequences were filtered to remove polyclonal, low quality, and adapter dimer, for a total of 32% of removed sequences. The remained 96,700,462 sequences, with an average length of 205 bp, were used to measure the relative abundances of the transcripts.

**FIGURE 1:**
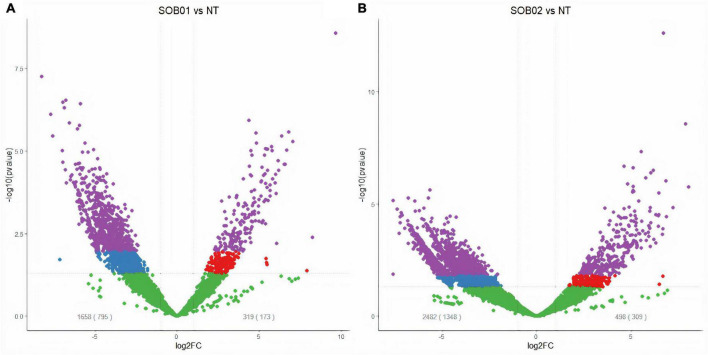
Volcano plots of significantly differentially expressed genes (DEGs) in tomato plants 48 h after treatment. Purple dots represent significantly up- and down-regulated DEGs (adj-*p* < 0.1 and | log2FC| > 1), red dots represent up-regulated DEGs (*p*-value < 0.05); blue dots represent down-regulated DEGs (*p*-value < 0.05); green dots are genes considered not differentially expressed according to the applied thresholds. Gray numbers display the number of DEGs according to the *p*-value threshold and in brackets according to the adj-p threshold. **(A)** DEGs in tomato plants 48 h after biostimulant treatment (SOB01) and non-treated (NT). **(B)** DEGs in tomato plants 48 h after calcium chloride treatment (SOB02) and non-treated (NT).

Totally, 968 significantly DEGs (adj-*p* < 0.1 and |FC| ≥ 2) were identified from the comparison between samples of SOB01 treated plants after 48 h from the product application and untreated plants before application (T0) (Figure 1A). Among the DEGs we identified 173 up-regulated genes and 795 down-regulated genes.

**FIGURE 2:**
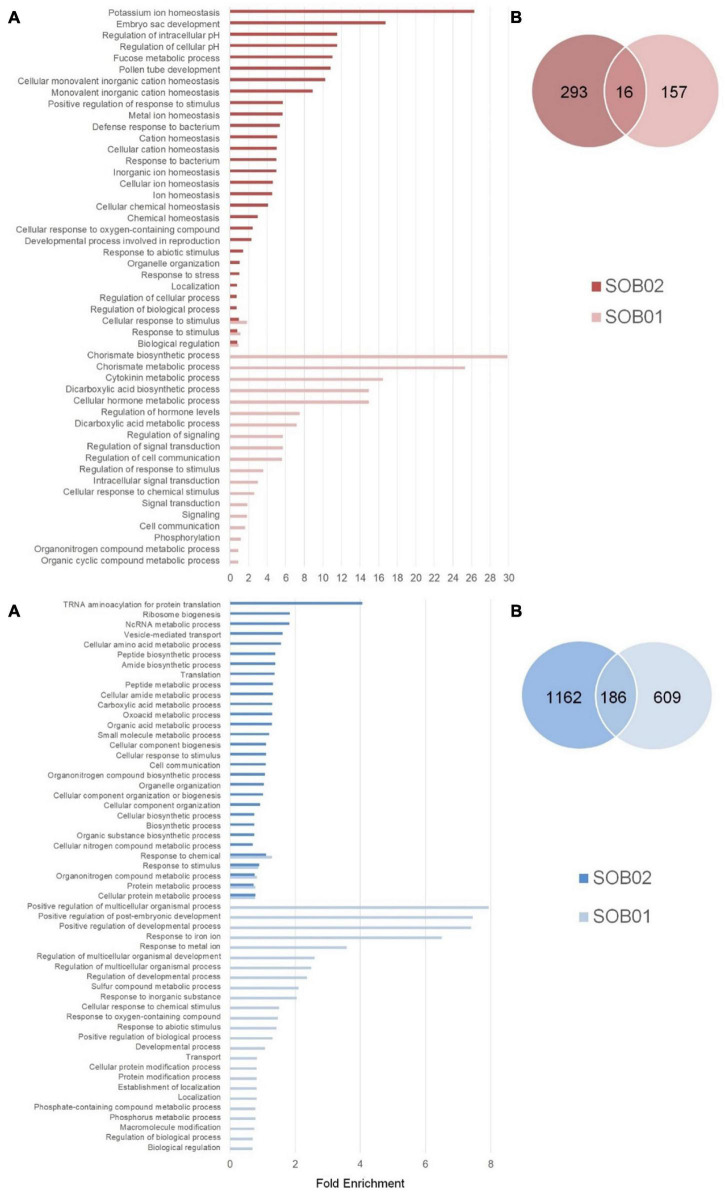
Categorization of DEGs (adj-*p* < 0.1 and | log2FC| ≥ 1) in tomato plants in response to SOB01 and SOB02 treatments. **(A)** Bar plots showing the significant enriched GO terms (FDR < 0.05), describing biological processes, for up-regulated DEGs (red) and down-regulated DEGs (blue). Results are obtained with ShinyGO online tool and *Solanum lycopersicum* as a reference genome. **(B)** Venn diagrams displaying the comparison of number of DEGs between SOB01 and SOB02. The number of uniquely DEGs are shown in the non-overlapping section and mutual DEGs among the two treatments in the overlapping section. Results for down-regulated genes (blue) and up-regulated genes (red) are shown in dark colors and light colors, respectively for SOB02 and SOB01 treatment in both Venn diagrams and bar plots.

The comparison between SOB02 and untreated plants showed 1,657 DEGs divided into 1,348 down-regulated and 309 up-regulated genes (Figure 1B). The comparison between the two treatments showed that 16 genes were significantly up-regulated by both treatments, 157 were uniquely up-regulated by SOB01, and 293 uniquely by SOB02 (Figure 2B, red diagram). Down-regulation was observed in 186 genes common to both treatments and 1,162 and 609 uniquely down-regulated respectively in SOB01 and SOB02 treated plants (Figure 2B, blue diagram).

Differentially regulated genes after SOB01 and SOB02 treatments were classified based on their functional category and a GO enrichment analysis was performed. The complete list of GO terms related to DEGs is given in Supplementary Tables 2, 3. Figure 2A is showing the significantly enriched GO terms related to biological process in response to SOB01 and SOB02 (FDR < 0.05). The “positive regulation of developmental and multicellular organismal processes” related GO terms were the main categories significantly enriched in the down-regulated genes after SOB01 treatment (Supplementary Table 2). Various “metabolic and biosynthetic process” GO terms, concerning peptides, organonitrogen compounds, organic substances, amino acids, were observed in the down-regulated genes in response to the calcium chloride standard fertilizer SOB02 alone.

The highest folds enrichment in up-regulated genes associated just with SOB01 were related with chorismate metabolism and regulation of hormone levels and signaling. Up-regulated genes in SOB02 treated plants were mostly related to ion homeostasis.

Among the significantly enriched gene categories commonly down-regulated in SOB01 and SOB02 treatments, were organonitrogen compound and protein metabolic processes and again “response to stimulus.” Whereas the few SOB01 and SOB02 commonly up-regulated genes encoded proteins involved in the cellular response to stimulus and biological regulation (Figure 2A).

### Quantitative Reverse Transcription PCR Assay Results

Nine genes selected within significantly enriched functional categories and emerged as relevant in the in-depth study of biostimulant applications were selected for further characterization of their expression on the three timings of treatment application and in WW and WS conditions. The selected genes are: *CRK*, *ABCI12*, *ERD15*, *NAC1*, *5203_PHOS32*, *3369_PHOS32*, *BOR02*, *SODCC1*, and *MRS2-4* (Table 1).

Real-time RT-qPCR was conducted on 48 samples divided into well-watered and water stress samples, two treatments, and three phenological stages. The experiments were repeated three times, and representative data are reported. In Table 2 relative expressions of the nine selected genes revealed significantly different expression levels between SOB01 and SOB02 for all the genes in at least one experimental condition (phenological stage or water regime).

**TABLE 2:** Relative expression levels of nine selected genes in tomato leaves after 48 h from the treatment application.

	Well-watered			Water stress		
	SOB01	SOB02	*p*-value	SOB01	SOB02	*p*-value
**Gene CRK**						
BBCH65	0.260 ± 0.150	0.260 ± 0.062	0.8380	0.300 ± 0.090	0.320 ± 0.110	0.6845
BBCH75	0.160 ± 0.035	0.130 ± 0.051	0.0871	0.120 ± 0.026	0.90 ± 0.032	0.0214 *
BBCH85	0.113 ± 0.040	0.111 ± 0.041	0.9536	0.095 ± 0.048	0.112 ± 0.051	0.3960
**Gene ABCI12**						
BBCH65	0.196 ± 0.011	0.182 ± 0.022	0.7216	0.248 ± 0.023	0.261 ± 0.088	0.4872
BBCH75	0.291 ± 0.024	0.275 ± 0.028	0.4015	0.300 ± 0.021	0.163 ± 0.015	0.0002 *
BBCH85	0.100 ± 0.097	0.072 ± 0.008	0.0032 *	0.095 ± 0.011	0.115 ± 0.009	0.1855
**Gene ERD15**						
BBCH65	0.045 ± 0.005	0.043 ± 0.005	0.7470	0.031 ± 0.006	0.038 ± 0.010	0.4377
BBCH75	0.068 ± 0.004	0.066 ± 0.006	0.9579	0.047 ± 0.006	0.056 ± 0.008	0.1156
BBCH85	0.100 ± 0.011	0.056 ± 0.004	0.0007 *	0.076 ± 0.012	0.082 ± 0.009	0.6534
**Gene NAC1**						
BBCH65	0.100 ± 0.008	0.069 ± 0.006	0.0044 *	0.080 ± 0.009	0.100 ± 0.010	0.1150
BBCH75	0.080 ± 0.005	0.078 ± 0.006	0.4367	0.078 ± 0.010	0.090 ± 0.010	0.2248
BBCH85	0.035 ± 0.002	0.022 ± 0.001	0.0001 *	0.026 ± 0.004	0.044 ± 0.003	0.0126 *
**Gene 5203_PHOS32**						
BBCH65	0.034 ± 0.004	0.033 ± 0.003	0.8324	0.020 ± 0.002	0.025 ± 0.006	0.4394
BBCH75	0.060 ± 0.005	0.050 ± 0.004	0.1926	0.055 ± 0.004	0.053 ± 0.007	0.6282
BBCH85	0.130 ± 0.020	0.080 ± 0.010	0.0078 *	0.180 ± 0.020	0.130 ± 0.010	0.0942
**Gene 3369_PHOS32**						
BBCH65	0.05 ± 0.002	0.06 ± 0.008	0.0938	0.005 ± 0.001	0.120 ± 0.013	0.0010 *
BBCH75	0.110 ± 0.003	0.120 ± 0.004	0.1590	0.082 ± 0.010	0.090 ± 0.010	0.6643
BBCH85	0.100 ± 0.020	0.060 ± 0.002	0.0009 *	0.090 ± 0.010	0.098 ± 0.009	0.5895
**Gene BOR.02**						
BBCH65	0.075 ± 0.005	0.037 ± 0.002	**0.00004**	0.060 ± 0.008	0.075 ± 0.010	0.3346
BBCH75	0.360 ± 0.100	0.390 ± 0.120	0.4083	0.390 ± 0.190	0.330 ± 0.290	0.0913
BBCH85	0.080 ± 0.007	0.045 ± 0.003	0.0006 *	0.050 ± 0.004	0.070 ± 0.008	0.0145 *
**Gene SODCC.1**						
BBCH65	0.0010 ± 0.0002	0.0014 ± 0.0009	0.0885	0.0015 ± 0.0004	0.0015 ± 0.0008	0.9700
BBCH75	0.0016 ± 0.0020	0.0011 ± 0.0010	0.1348	0.0007 ± 0.0002	0.0024 ± 0.0021	0.0265 *
BBCH85	0.0017 ± 0.008	0.0015 ± 0.0005	0.6066	0.0024 ± 0.0006	0.0024 ± 0.0010	0.9514
**Gene 7864_MRS2-4**						
BBCH65	0.040 ± 0.005	0.055 ± 0.004	0.0276 *	0.035 ± 0.002	0.090 ± 0.011	0.0149 *
BBCH75	0.250 ± 0.030	0.230 ± 0.070	0.4788	0.240 ± 0.020	0.267 ± 0.031	0.1730
BBCH85	0.210 ± 0.018	0.200 ± 0.005	0.2298	0.250 ± 0.070	0.280 ± 0.020	0.2690

Data are means ± S.D. and p-values of Student t-test comparisons (p < 0.05) between SOB01 and SOB02 in the two water regimes (WW and WS). Significantly different means (p < 0.05) are marked with a * and respective p-values are written in bold.

**FIGURE 3:**
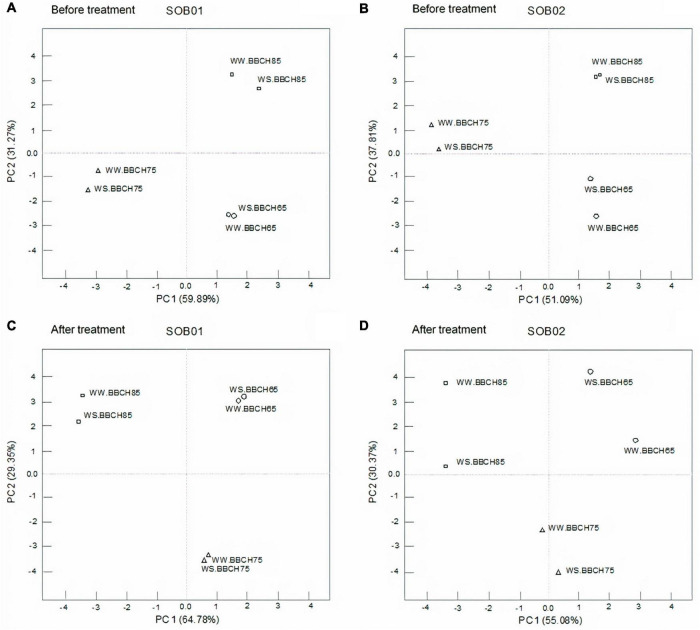
Principal component analysis of gene expression data of well-watered (WW) and water stress (WS) samples before treatment **(A,B)** and after treatment **(C,D)** with SOB01 **(A,C)**, and SOB02 **(B,D)** in three different phenological stages (BBCH65, BBCH75, and BBCH85).

Figure 3 shows the results of the principal component analysis (PCA) accomplished on gene expression data from RT-qPCR, pooling the results of 24 WS and 24 WW samples, each group composed of 12 plants treated with SOB01 and 12 treated with SOB02, and divided by three phenological phases. Figures 3A,B display the PCs obtained from gene expression data divided by phenological stages before SOB01 and SOB02 treatments, respectively. Figures 3C,D show the effect after SOB01 and SOB02 application, respectively, in the three phenological stages on WW and WS samples. PCA is often used in exploratory data analysis and pattern recognition as a tool to highlight differences among several types of samples.

The PCAs in Figure 3 distinguish different groups based on phenological stage for each different sampling time. WW and WS in the different plant stages are clustering together after SOB01 treatment (Figure 3C), whereas gene expression data of WW and WS plants after treatment with the standard fertilizer SOB02 are not clustering together (Figure 3D).

### Physiological Responses to Biostimulant Application and Water Stress

To assess the physiological responses of tomato plants to water deficit and biostimulant application, physiological traits including tomato fruit dry matter, number of cracked fruits, leaf gas exchange parameters, chlorophyll fluorescence, and SPAD were evaluated.

The physiological measurements taken in BBCH65 showed no significant effects of the treatments. A significant interaction was observed between the fertilizers and water regimes only at the fruit development phase (BBCH75). Although, there is no significant interaction between the two factors at BBCH65 and BBCH85 (Table 3). Except for the net photosynthesis rate, the water regime never influenced the plant response to fertilizers treatments.

**TABLE 3:** Analysis of variance of the physiological measured parameters that were affected by foliar application of fertilizers (F) and the Water regimes (W) at three phenological phases.

	BBCH											
	65				75				85			
	gs	A	Fv/Fm	SPAD	gs	A	Fv/Fm	SPAD	gs	A	Fv/Fm	SPAD
Fertilizer (F)	ns	ns	ns	ns	*	*	ns	*	*	ns	ns	ns
Water regime (W)	ns	ns	ns	ns	ns	ns	ns	ns	ns	ns	ns	ns
F × W	ns	ns	ns	ns	ns	*	ns	ns	ns	ns	ns	ns

*SOB01, novel calcium-based biostimulant. SOB02, calcium-chloride fertilizer. A, net photosynthesis. Gs, stomatal conductance. Fv/Fm, chlorophyll fluorescence. BBCH65 (5th inflorescence), BBCH75 (5th fruit cluster), BBCH85 (50% of fruits show typical fully ripe color).*

*ns, non-significant.*

*Significant at the 0.05 level of probability.

**FIGURE 4:**
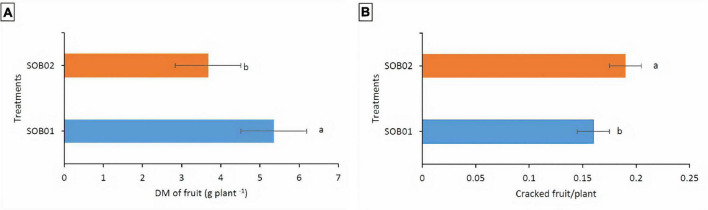
Average tomato fruit dry matter **(A)** and number of cracked fruits **(B)** per plant in response to different treatments. SOB01, novel calcium-based biostimulant. SOB02, calcium-chloride fertilizer. DM, dry matter. Different letters indicate a significant difference according to LSD test (*p* < 0.05).

Results indicated that the water regime did not affect the response of the fertilizers treatment on fruit dry matter content and the number of cracked fruits. In general, tomato plants treated with SOB01 showed significantly higher fruit dry matter production (5.35 g plant^–1^) than those treated with SOB02 fertilizer (3.67 g plant^–1^) (Figure 4A). Furthermore, the biostimulant treatment decreased the rate of cracked fruits (0.16 fruit plant^–1^) compared to SOB02 fertilizer (0.19 fruit plant^–1^) (Figure 4B).

**FIGURE 5:**
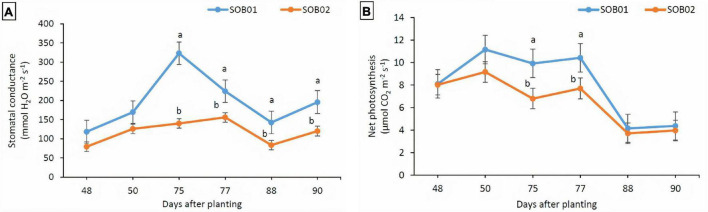
Effect of foliar fertilizer SOB01 and SOB02 on stomatal conductance **(A)** and net photosynthesis **(B)** at different phenological stages of tomatoes. Physiological measurements during plant growth were carried out before the application and 48 h after treatments application. SOB01, novel calcium-based biostimulant. SOB02, calcium-chloride fertilizer. Phenological stages BBCH65 (5th inflorescence = flowering), BBCH75 (5th fruit cluster = fruit development), BBCH85 (50% of fruits show typical fully ripe color = maturity). Different letters indicate a significant difference according to LSD test (*p* < 0.05).

The positive effects of SOB01 in terms of stomatal conductance and net photosynthesis were evident 48 h after the application at BBCH75 and BBCH85. The leaf stomatal conductance detected on plants treated with SOB01 was significantly higher than the one detected on plants treated with SOB02 before and after the second (BBCH75) and the third (BBCH85) application (Figure 5A). Stomatal conductance in plants treated with SOB01 reached its maximum value earlier in time compared to what was observed in plants treated with calcium chloride SOB02. Moreover, the net photosynthesis rate on plants treated with SOB01 was significantly higher than SOB02 treatment only at BBCH75, before and after application (Figure 5B).

**FIGURE 6:**
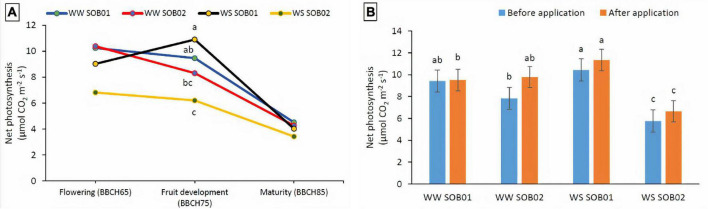
Interactions between different water and fertilizer treatments on net photosynthesis. **(A)** At different phenological stages and **(B)** at the fruit development stage (BBCH75) before and after treatment application. Measures were taken before the treatment application and 48 h after application. SOB01, novel calcium-based biostimulant. SOB02, calcium-chloride fertilizer. WW, well water. WS, water stress. Different letters indicate a significant difference according to LSD test (*p* < 0.05).

A significant interaction between the different fertilizers and water regimes was detected on net photosynthesis at the fruit development stage (BBCH75) only (Figure 6). The application of the novel calcium-based biostimulant on plants partially compensated for the effect of water deficiency on net photosynthesis. The net photosynthesis of stressed plants treated with SOB01 showed almost a 2-fold increase compared to the plants treated with calcium-chloride fertilizer (SOB02) in the same water deficit conditions. Similar patterns were observed for well-watered plants (Figures 6A,B).

**FIGURE 7:**
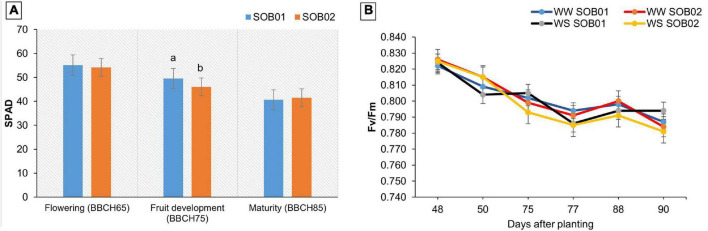
Soil Plant Analytical Division (SPAD) value **(A)** and Fv/Fm (maximum potential quantum efficiency of PSII) **(B)** in leaves of tomato at different phenological stages. Measures were taken before the application and 48 h after treatment application. SOB01, novel calcium-based biostimulant. SOB02, calcium-chloride fertilizer. WW, well water. WS, water stress. Different letters indicate a significant difference according to LSD test (*p* < 0.05).

Physiological surveys performed during the plant growth showed significant effects of the treatment on the SPAD values. No significant changes in plant SPAD value were observed after treatments application at flowering and maturity stages. Conversely, the novel biostimulant (SOB01) induced a significant increase in this parameter, after the application at BBCH75 (fruit development stage) (Figure 7A). In contrast, no significant response of plants’ chlorophyll fluorescence (Fv/Fm) to SOB01 and SOB02 application was observed at any developmental stage (Figure 7B).

## Discussion

Plant biostimulants constitute an emerging class of agricultural inputs that help to improve crop yield and quality while protecting from biotic and abiotic stresses (Rajput et al., 2019). The effects are dependent on application and dosage and act differently on different cultivars and environmental conditions (Di Mola et al., 2019). The ability to predict plant response to biostimulants is a high priority in the development of sustainable agriculture.

In this study, the applications of a novel calcium-based biostimulant (SOB01) and a calcium-chloride fertilizer (SOB02) were compared to elucidate their different effects on tomato plants under well-watered and water stress conditions. Since biostimulants have broad-spectrum activity, involving many plants’ metabolic pathways, we initially focused on the analysis of the plant’s transcriptome. This analysis allows the identification of changes in gene expression providing evidence regarding the pathways and the biological processes involved in the treatment-induced responses.

To describe all the effects produced by the biostimulant, we identified DEGs among plants before and after treatment with SOB01 and SOB02. The analysis output only 16 commonly up-regulated genes, indicating a dissimilarity in the transcriptome modulation after the fertilizer and the biostimulant application. For both the products, the number of down-regulated genes was greater than the number of up-regulated ones.

We then identified a panel of nine genes belonging to nutrient transport and metabolism and involved in the response to osmotic and water stress. The extensive characterization of the expression levels of the selected genes in the three different phenological stages corresponding to the treatment application times, on well-watered and water-stressed samples, was carried out. The PCA plot is highlighting how the expression data of these nine genes in WW and WS plants after treatment with the standard fertilizer SOB02 are not clustering together in the same phenological stage. This pattern of expression could suggest that the applied water stress or the treatment SOB02 caused a variation in the expression of the nine genes, leading to the separation of WS samples from the WW ones.

We observed that two genes involved in chorismate metabolism were up-regulated and significantly enriched in SOB01 treated plants. Chorismic acid represents a key step in the shikimate pathway of aromatic acid biosynthesis, being a precursor to the aromatic amino acids phenylalanine, tyrosine, and tryptophan (Maeda and Dudareva, 2012). Being crucial for the synthesis of aromatic amino acids and other secondary metabolites, the gene encoding the 5-enolpyruvylshikimate-3-phosphate synthase (EPSPS), is fundamental for plant growth and development. Chorismate is also a common precursor of the three phytohormones auxin, salicylic acid, and melatonin (Pérez-Llorca et al., 2019). This observation could be explained as an activation of the plant signaling pathway in response to the application of SOB01 and its sugar fraction in particular. It is known that oligo- or polysaccharides are signaling molecules acting as elicitors of several pathways involved in plant secondary metabolisms (Zhao et al., 2005). This possibility is further reinforced by the observed significant enrichment associated with SOB01 up-regulation of the biological process described as “regulation of signal transduction” in which a gene coding for an ethylene receptor is over-expressed.

In the leaves treated with SOB01 we also observed an up-regulation of two genes involved in the cytokinin metabolic process which had a significant fold enrichment. Specifically, the gene LOG3 encoding a cytokinin riboside 5′-monophosphate phosphoribohydrolase is converting inactive cytokinin nucleotides to their biologically active forms. Cytokinins are a class of purine-based molecules with hormonal activity in plants which is promoting not only cell division and differentiation, but also growth, delay of senescence, and protection from oxidative stress (Mok and Mok, 2001). Cytokinins at leaf-level affect in several ways the photosynthetic process by promoting cell differentiation, increasing stomatal conductance, and improving the number and differentiation of chloroplasts (Hönig et al., 2018). The upregulation of key genes involved in the cytokinins activation following SOB01 treatment is positively correlated with the higher stomatal conductance measured in plants treated with SOB01 compared with the ones treated with SOB02. Moreover, the protection of the photosynthetic machinery potentially exerted by an increased cytokinins activation in the leaves, together with the antioxidant activity of these phytohormones, could have played a role in the overall mitigation of drought stress-induced detrimental effects.

Among the DEGs, obtained by the RNA-Seq analysis, down-regulated by SOB01 application we found SODCC.1 (*Solyc01g067740*) that encodes a Cu-Zn superoxide dismutase. It belongs to the significantly enriched GO terms of response to abiotic stimulus, chemical, metal ions, and biological regulation. It was not differentially expressed in SOB02 treated plants. In the RT-qPCR analysis validation, the expression level of the same gene was significantly lower (3.4-folds lower) in SOB01 treated plants, sampled at the 5th fruit cluster (BBCH75) in water stress, compared to SOB02. Water stress is known to cause a wide range of plant responses. One of the most important is the increase in oxidative stress (Rao and Chaitanya, 2016). Superoxide dismutases (SODs) are a group of antioxidant metalloenzymes that protect cells from oxidative stress by catalyzing the dismutation of superoxide radicals to molecular oxygen and hydrogen peroxide. To regulate the ROS levels and restore normal physiological status, variations in the expression of SOD-encoding genes in response to environmental stresses are expected (Wang et al., 2016). Specifically, in young tomato plants, the expression of *Solyc01g067740* gene was previously evaluated by Feng et al. (2016) in response to high salinity and polyethylene glycol-induced drought stress. The former stress induced significant up-regulation of SODCC.1, whereas in drought conditions the level of expression did not change with respect to the control. In general, the activity of ROS scavenger enzymes in plants increases as a response to drought stress (Das and Roychoudhury, 2014). Moreover, responses to biostimulants involving the dysregulation of the superoxide dismutase gene family were observed in several works. Biostimulant application has been observed to either promote this antioxidant enzymatic activity as a response to the water-limited conditions (Liu et al., 2013) or, even before the stress application, exerting a priming effect that restrains the negative effects of the incoming stress (Goñi et al., 2016; Santaniello et al., 2017). In other cases, it can conversely slow down the activation of such metabolic pathways, compared to what happens in untreated plants, suggesting a process of adaptation to the drought stress due to the biostimulant treatment (Murtic et al., 2019; Campobenedetto et al., 2021). Our results on the lower expression levels of SODCC.1 in water-stressed plants treated with the novel biostimulant, compared to plants treated with the standard CaCl2 fertilizer, are following these last observations. Anyway, several studies on calcium chloride applications have shown the capacity of this mineral fertilizer to induce increased activities and gene expressions of superoxide dismutase and catalase which are protecting from oxidative stress produced by cold, pathogens, and drought (Xu et al., 2013; Shi et al., 2014; Chakraborty et al., 2017; Hou et al., 2021). Nevertheless, we did not observe a significant up-regulation of superoxide dismutase following SOB02 treatments in WW conditions and the physiological results in our experiment seem to encourage the interpretation of the lower expression of SODCC.1 gene in SOB01 plants as a reduction of the water stress susceptibility of the plants.

The application of the novel calcium-based biostimulant (SOB01) enhanced dry matter yield and fruit quality. The dry matter content of tomato plants treated with SOB01 was 50% significantly higher than the one of plants treated with calcium chloride (Figure 4A). Furthermore, the biostimulant treatment decreased the rate of cracked tomato fruits per plant compared to SOB02 treated plants (Figure 4B). Tomato fruit cracking is a serious problem that results in significant financial losses. The fruit development rate during the ripening stage, maybe sustained by internal turgor pressure, is a key factor in fruit cracking (Domínguez et al., 2012). Fruit cracking can occur during fruit growth and/or ripening time. The cracking of the fruit is the result of a physiological imbalance determined by the action of multiple factors and physical nature linked to the plant. In addition to genetic susceptibility, water stress is one of the main determinants of cracking. Therefore, it is likely that fruit cracking was reduced in SOB01 treated plants through the positive effects on drought tolerance that allowed sustaining the plant during fruit development (Chrysargyris et al., 2020; Petropoulos et al., 2020).

Enhanced photosynthetic efficiency and a greater level of plant water content under drought stress conditions indicate an improved metabolic activity of plants (Figures 6A,B). In our experiment, it is likely that the biostimulant improved plant water status under drought and promoted cell enlargement, preventing ROS damage to pollen viability, with a beneficial effect on fruit development. Drought stress in plants leads to inhibition of photosynthesis and respiration, accumulation of ROS, and reprogramming of gene expression (Selote et al., 2004; Hayano-Kanashiro et al., 2009; Meng et al., 2020). The improved water status and the protection of cellular membranes under drought could be the reason for the higher yield reported in plants treated with SOB01, which was mediated by the higher drought tolerance of these plants during the sensitive stages of fruit development (BBCH75) and enlargement (Figure 6; Francesca et al., 2021). Additionally, our results on leaf gas exchange (Figure 5) were consistent with the findings of other researchers (Colla et al., 2017b; Parađiković et al., 2019; Soppelsa et al., 2019; Francesca et al., 2021), who observed that biostimulants application can enhance the leaf gas exchange characteristics to maintain plant water status under the water deficiency, improve nutrient uptake in plants, promote plant vigor and uniformity, be effective in regulating flowering, and stimulate fruit set and ripening.

Of the many biological processes activated when plants encounter environmental stresses, the photosynthesis-related processes and gas exchange responses are the most sensitive to water deficit (Hayano-Kanashiro et al., 2009; Huo et al., 2016; Min et al., 2016). Since photosynthesis is one of the main physiological processes affected by drought, photosynthetic parameters have been universally used to evaluate plant drought tolerance (Chaves et al., 2009; Osakabe et al., 2014; Zhang et al., 2018; Zhao et al., 2020).

Another common physiological response in plants suffering from drought stress is stomata closure. The closing of stomata is a well-known mechanism that plants use to avoid water loss in response to drought stress (Yan et al., 2016), but this adaptation also results in decreased CO_2_ flux (Ying et al., 2012). As water stress continues, the stomata remain closed for longer during the day. This leads to a reduction in carbon assimilation rate and water loss, which results in the maintenance of carbon assimilation at the expense of low water availability (Zhao et al., 2020).

The stomatal limitation is generally considered a major factor in the weakening of photosynthesis under water stress (Jones, 1998; Tardieu and Simonneau, 1998). In the case of water deficit, the reduction of leaf relative water content and water potential causes the stomata to close, leading to a decrease in the effectiveness of CO_2_ and net photosynthesis (Bota et al., 2004; Zhao et al., 2020). Scientific evidence shows that photosynthesis, photochemical efficiency, and gas exchange processes are significantly less affected by stresses when biostimulants are applied (Van Oosten et al., 2017).

In this research, stomatal conductance was affected by the treatment application at fruit development and ripening stages, but stomatal closure was not significantly affected by water stress, resulting in no obvious reduction in photosynthesis. On the other hand, net photosynthesis was significantly affected by water stress and increased with the application of SOB01 at the fruit development stage (Figures 6A,B). This significant biostimulant-induced enhancement in photosynthesis of plants grown under WS conditions may be attributed to the changes in the photosynthetic machinery, chlorophyll content, leaf area, temperature, and leaf relative water content. These data are consistent with previous reports by Xu and Leskovar (2015) and Kałużewicz et al. (2017), who concluded that biostimulant application improved leaf water relations and helped to maintain cell turgor pressure and reduced stomatal closure, increased photosynthetic rate, and consequently enhanced growth. Furthermore, this seems to be consistent with the PCAs of genes expression results which show that, after the application at BBCH75, plants treated with SOB01 have a similar expression profile both in conditions of correct irrigation and under a reduced water regime (Figure 3C).

The positive effect of plant biostimulants is also based on increasing the content of chlorophyll in leaves and thus increasing the efficiency of photosynthesis. Chlorophyll is the main pigment carrying out photosynthesis in plants, involving the process of light energy absorption, transfer, distribution, and transformation (Biswal et al., 2011). The decrease in PSII photochemical efficiency in environmental stress conditions may be related to a reduction of chlorophyll content (Song et al., 2014). Indeed, this index is considered a major indicator of green pigment biosynthesis efficiency and thus improved crop yields (Di Mola et al., 2019). In the current study, leaf SPAD measurements recorded in biostimulant-treated plants were significantly higher than in calcium chloride-treated plants after the flowering stage, which might contribute to the improved photosynthetic rate of tomato leaves under water deficit. The higher SPAD values may have been caused by different mechanisms: (i) increased N uptake efficiency, (ii) reduced chlorophyll degradation and leaf senescence, and (iii) modified hormonal metabolism (Jannin et al., 2013; Bhattacharyya et al., 2015; Ertani et al., 2017). Different phytohormones are involved in leaf senescence and stomatal conductance (Luo et al., 2019). We have previously mentioned how the role of cytokinins, acting as antagonists of abscisic acid, can delay leaf senescence and promote gas exchanges at leaf level (Hönig et al., 2018). Hormone metabolic process and regulation of hormone levels are among the enriched GO terms category of up-regulated genes after SOB01 application (Figure 2A). Therefore, the increased stomatal conductance and SPAD value measured in plants treated with the biostimulant can be caused by an alteration of the hormone profile (Russell et al., 2006).

## Conclusion

The main goal of this preliminary research was to provide a rigorous multidisciplinary approach to the characterization of the activity of a plant biostimulant, using tomato (*S. lycopersicum* L.) as a model crop.

Transcriptomics and physiological analyses have provided a detailed description of the different modes of action exerted by the biostimulant product compared to a classic fertilizer both in water stress and well-watered conditions.

At the molecular level, the modulation of different genes categories both in terms of up-regulation and down-regulation by the biostimulant compared to the standard calcium-chloride fertilizer suggests a peculiar activity exerted by the novel product. Furthermore, the mitigation of some stress-related genes detected on plants treated with the biostimulant could explain the observed improved physiological parameters in plants subjected to water stress.

Consistent with this, physiological measurements demonstrated that biostimulant application increased the photosynthetic rate and the chlorophyll content under water deficiency, helping the plant to cope with drought and resulting in the higher production of fruit dry matter and reduction of cracked fruits. Moreover, the biostimulatory action of the new calcium-based biostimulant resulted in improved stomatal response at tomato fruit development and ripening stage.

To validate this multidisciplinary approach for the characterization of the plant biostimulant activity at different levels of environmental and genetic variability, further studies are required.

## Data Availability Statement

The datasets presented in this study can be found in online repositories. The names of the repository/repositories and accession number(s) can be found below: ENA–ERX6700091-ERX6700098.

## Author Contributions

FM, PS, and AM: conceptualization and supervision. FM, PS, AM, GC, MD, AB, MB, WZ-L, SR, SD, and CB: methodology. AB, CB, MD, and PS: writing the original draft. FM and SN: writing, review, and editing. All authors contributed to the article and approved the submitted version.

## Conflict of Interest

FM is employed by Sipcam Italia S.p.A. belonging together with Sofbey SA to the Sipcam Oxon S.p.A. Group (Pero, Italy). The remaining authors declare that the research was conducted in the absence of any commercial or financial relationships that could be construed as a potential conflict of interest.

## Publisher’s Note

All claims expressed in this article are solely those of the authors and do not necessarily represent those of their affiliated organizations, or those of the publisher, the editors and the reviewers. Any product that may be evaluated in this article, or claim that may be made by its manufacturer, is not guaranteed or endorsed by the publisher.

## Footnotes

1
http://bioinformatics.sdstate.edu/go/
