# Supplementary material for: Transcriptional and Physiological Analyses to Assess the Effects of a Novel Biostimulant in Tomato
Source: Front Plant Sci. 2022 Jan 11;12:781993. doi: 10.3389/fpls.2021.781993 (PMC8787302; doi:10.3389/fpls.2021.781993)

Supplementary Material

Supplementary Figures

Supplementary Figure 1. Figure S1A (**A**) reports the electropherogram obtained with Agilent TapeStation 1500 of one mRNA sample (1,930 pg/µl) showing a contamination from 18S and 28S sequences while Figure S1B (**B**) shows the electropherogram of the same tomato mRNA sample after the additional washing step. Figure S1C (**C**) and S1D (**D**) display the electropherogram of fragmented mRNA and of the final obtained cDNA library, respectively.

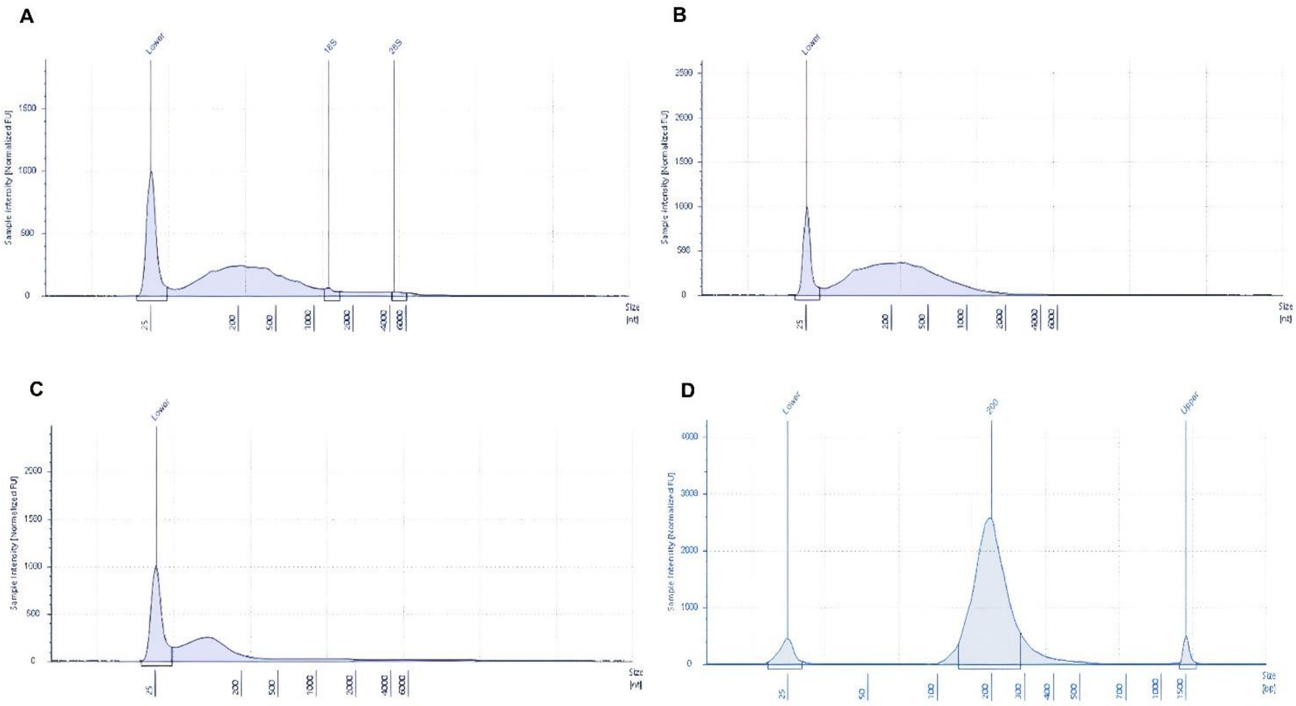

Supplementary Figure 2. Chip loading and sequencing details from Ion S5 server.

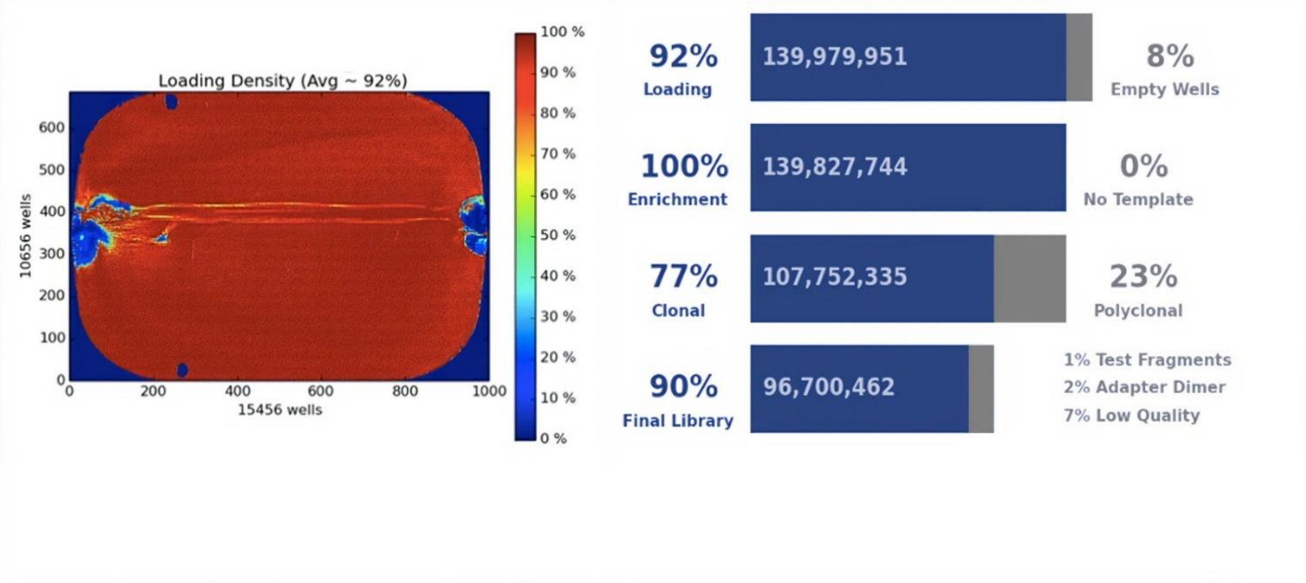

Supplement: Supplementary file 1 [file Data_Sheet_1.pdf]
