# Supplementary material for: Transcriptional and Physiological Analyses to Assess the Effects of a Novel Biostimulant in Tomato
Source: Front Plant Sci. 2022 Jan 11;12:781993. doi: 10.3389/fpls.2021.781993 (PMC8787302; doi:10.3389/fpls.2021.781993)
Supplement: Supplementary file 2 [file Table_1.DOCX]

Supplementary Material

## Supplementary Tables

**Supplementary table 1.** Chemical composition and properties of the calcium-based biostimulant SOB01 provided by Sipcam-Oxon S.p.a.

| CaO sol. in water (% w/w) | 5 |
| --- | --- |
| Mn sol. In water (% w/w) | 1.5 |
| Zn sol. In water (% w/w) | 0.5 |
| Saccharides mixture* (% w/w) | 20 |
| pH | 4.25 |
| Electrical conductance (mS/cm) | 28.8 |
| Density (kg/l) | 1.407 |

*composed by monosaccharides (30.53%), disaccharides (26.02%), polysaccharides (20.48%).
